# Pteropods are excellent recorders of surface temperature and carbonate ion concentration

**DOI:** 10.1038/s41598-017-11708-w

**Published:** 2017-10-03

**Authors:** N. Keul, K. T. C. A. Peijnenburg, N. Andersen, V. Kitidis, E. Goetze, R. R. Schneider

**Affiliations:** 1 Institute of Geosciences, Christian-Albrechts-Universität zu Kiel, Ludewig-Meyn-Str.10, 24118 Kiel, Germany; 2 Naturalis Biodiversity Center, P.O. Box 9517, 2300 RA Leiden, The Netherlands; 3 Institute for Biodiversity and Ecosystem Dynamics (IBED), University of Amsterdam, P.O. Box 94248, 1090 GE Amsterdam, The Netherlands; 4 Leibniz-Labor für Altersbestimmung und Isotopenforschung, Christian-Albrechts-Universität zu Kiel, Max-Eyth-Str.11–13, 24118 Kiel, Germany; 5 Plymouth Marine Laboratory, Plymouth, PL1 3DH United Kingdom; 6 Department of Oceanography, University of Hawai’i at Mānoa, 1000 Pope Road, Honolulu, HI 96822 USA

## Abstract

Pteropods are among the first responders to ocean acidification and warming, but have not yet been widely explored as carriers of marine paleoenvironmental signals. In order to characterize the stable isotopic composition of aragonitic pteropod shells and their variation in response to climate change parameters, such as seawater temperature, pteropod shells (*Heliconoides inflatus*) were collected along a latitudinal transect in the Atlantic Ocean (31° N to 38° S). Comparison of shell oxygen isotopic composition to depth changes in the calculated aragonite equilibrium oxygen isotope values implies shallow calcification depths for *H*. *inflatus* (75 m). This species is therefore a good potential proxy carrier for past variations in surface ocean properties. Furthermore, we identified pteropod shells to be excellent recorders of climate change, as carbonate ion concentration and temperature in the upper water column have dominant influences on pteropod shell carbon and oxygen isotopic composition. These results, in combination with a broad distribution and high abundance, make the pteropod species studied here, *H*. *inflatus*, a promising new proxy carrier in paleoceanography.

## Introduction

Assessing the future impact of ocean acidification, the decline in oceanic pH due to anthropogenic CO_2_ emissions, on marine ecosystems is difficult, as the complexity of ecosystems cannot be easily replicated in laboratory experiments. However, long-term evidence for ocean acidification and the associated responses of marine calcifiers can be found in the geological record, as past ocean temperature and chemistry can be derived from fossil calcium carbonate shells, as well as ecosystem responses such as species richness and net calcification. One prominent and straightforward candidate for such an approximation (proxy) of past conditions is the oxygen isotopic composition (δ^18^O), which reflects ocean temperature^1^. It has been demonstrated in inorganic precipitation studies as well as in direct measurements of biogenically produced calcium carbonate^2,3^, that the calcium carbonate- water oxygen isotopic equilibrium is determined by temperature and (sea)water δ^18^O (δ^18^O_SW_; see Table 1 for notations). This observation applies to both calcite^4,5^ and aragonite^3,6^, with an aragonite-calcite fractionation on the order of 0.7 to 0.9‰^7^. Most studies have analyzed the calcite produced by foraminifera in this context. Similar to foraminifera, pteropods secrete aragonite close to the calcium carbonate- seawater δ^18^O equilibrium^6,8^, making them ideal candidates to study their oxygen isotopic composition. Pteropods are marine holoplanktonic gastropods inhabiting epipelagic and mesopelagic waters, down to >1000 m depths^9^. Although pteropods are rarely explored as possible oceanographic proxy carriers compared to foraminifera, several aspects of their biology make them interesting targets. The approximately one year life cycle and diel vertical migrations of pteropods may yield a more integrative proxy record across epipelagic and mesopelagic water masses and across seasons in comparison to foraminifera, which are characterized by a shorter life span and potentially shallower calcification depths. Calcification depths of recent pteropods can be estimated by comparing the oxygen isotopic composition to the theoretical δ^18^O of aragonite precipitated in equilibrium with the seawater (δ^18^O_ara_), which is calculated from temperature profiles and δ^18^O_SW_. Temperature at the time of calcification can be reconstructed directly from δ^18^O_ptero_, the oxygen isotopic composition of pteropod shells (recent or fossil).

**Table 1:** Notations.

DIC	dissolved inorganic carbon
δ^18^O_SW_	δ^18^O of seawater
δ^18^O_ara_	theoretical δ^18^O of aragonite precipitated in equilibrium with the seawater
δ^18^O_ptero_	δ^18^O of pteropod shells
δ^13^C_DIC_	δ^13^C of DIC in seawater
δ^13^C_ptero_	δ^13^C of pteropod shells

δ^13^C in calcium carbonate shells is assumed to be in equilibrium with, or offset by a constant amount from δ^13^C_DIC_, the carbon isotopic composition of dissolved inorganic carbon (DIC; the sum of CO_2(aq)_, H_2_CO_3_, HCO_3_^−^ and CO_3_^2−^). However, several studies found discrepancies for other groups of calcifiers, e.g. foraminifera^10^, as physiological processes, such as the respiration of symbionts in foraminifera, influence shell δ^13^C^11^. Juranek and colleagues^12^ found a correlation between pteropod δ^13^C_ptero_ and carbonate ion concentration and hypothesized that this was caused either by a carbonate ion dependence, also demonstrated for foraminifera^13^, or by the influence of temperature on metabolic CO_2_ incorporation.

The life cycle of the pteropod species *Heliconoides inflatus* (d’Orbigny, 1834^14^), formerly and more commonly known as *Limacina inflata*, has been estimated to be approximately 7–9 months, with a maximum life span of about one year^15,16^. Reproduction occurs continuously throughout the year, with females retaining developing embryos in the mantle cavity up to a size of about 70 μm until release of the veliger larvae^15^. Sediment trap studies revealed high interannual variability in the abundance of *H*. *inflatus* as well as a pronounced seasonal cycle in abundance, e.g. in the Sargasso Sea^16^. Here, the highest flux of shells was found during summer, whereas the flux throughout the rest of the year was very low^16,17^. While *H*. *inflatus* has been found as deep as 1000 m, this species primarily occurs in the upper water column^12^. Seasonal shifts in pteropod depth habitat were also reported for the Sargasso Sea, with *H*. *inflatus* preferring shallower waters in the fall (100 to 250 m) than in the spring/ early summer (200–400 m)^9^. Diel vertical migration, common in pteropods, has been described for *H*. *inflatus*, with most of the population below 200 m during the day and﻿ highest night-time abundance in the upper 75m^9^. The species also undergoes ontogenetic migration: juveniles tend to stay in surface waters, whereas adults are mainly found in deeper waters^17^.

Here we present δ^18^O_ptero_ and δ^13^C_ptero_ measurements of *Heliconoides inflatus* shells to assess the potential of pteropods to serve as proxies of seawater temperature (via δ^18^O) and carbonate ion concentration (via δ^13^C). We particularly focus on the calcification depth of the species and characterize environmental controls on shell composition. Our material was collected along a meridional transect in the Atlantic Ocean, ranging from 31° N to 38° S. This provides an unique opportunity to assess calcification depths across the entire Atlantic basin, in contrast to previous sediment trap studies that were conducted within a single oceanographic context^12,16,18,19^. Furthermore, the broad spatial scope of the study allows us to assess different environmental parameters as controls on the stable isotopic composition in pteropod shells, as oceanographic conditions change significantly over such a large latitudinal range. The calibrations established here will be of use to the ocean acidification and paleo-oceanographic community, as the studied species, *H*. *inflatus*, occurs in high abundance in sediments worldwide^20^, for instance, in the Central and South Atlantic^21^ or the Caribbean Sea^22^.

## Results

### Surface distribution of oceanographic parameters

**Figure 1:**
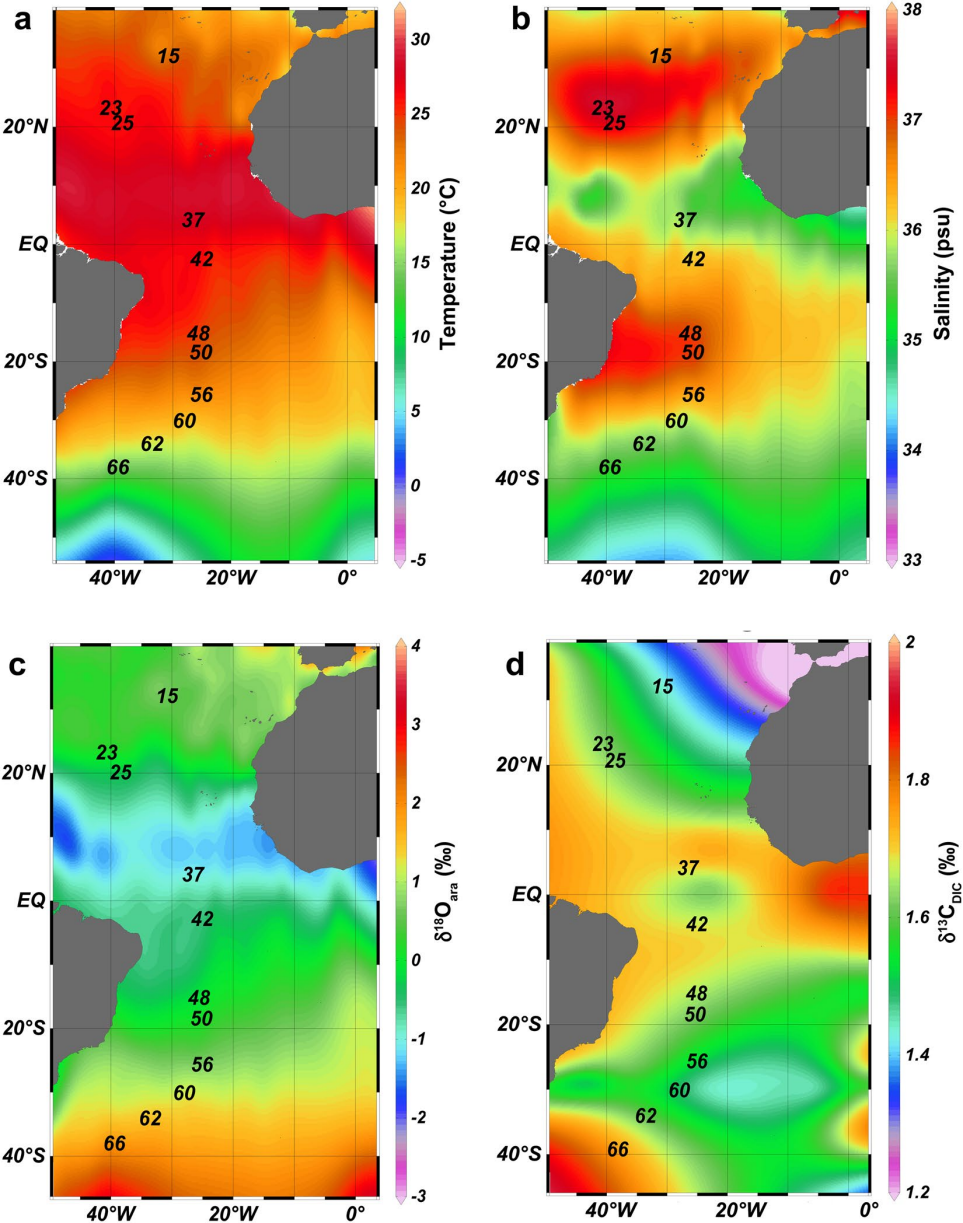
Distribution plots of hydrographic parameters in surface waters in the Atlantic Ocean. (**a**) temperature (° Celsius), (**b**) salinity, (**c**) δ^18^O_ara_, and (**d**) δ^13^C_DIC_. Only stations from which pteropods were sampled for this study are shown. Data for panels (**a**), (**b**) and (**c**) are derived from the World Ocean Database^38^, data for panel (**d**) are from GLODAP^39^. The software Ocean Data View (v. 4.6.3, http://odv.awi.de) was used to generate these maps^40^.

The warmest surface temperatures in the study area of the Atlantic Ocean, up to ~30 °C, usually occurred in October/November just north of the equator (around 10° N; Fig. 1a). Temperature decreased gradually both north and south of this maximum to approximately 12 °C at 40° S, where our southernmost station (station 66) was located. The surface salinity distribution mimicked this general latitudinal zonation (Fig. 1b), however, at the latitude of the temperature maximum, low salinities of 35 to 36 prevailed. Highest surface salinities (37–37.5) occurr﻿ed in two locations west of 30° W: one in the North Atlantic at around 25° N and one in the South Atlantic at around 20° S. The three southernmost stations (stations 60, 62, 66) lie in an area characterized by mean salinities between 35 and 36. Theoretical values for δ^18^O of aragonite (δ^18^O_ara_), taking into account δ^18^O estimates of seawater and ambient temperatures, as well as values for δ^13^C_DIC_ were calculated for surface waters (Fig. 1c, d; see Methods and Table 1 for notations). The strong influence of temperature on the surface distribution of δ^18^O_ara_ is evident from the latitudinal zonation of this parameter (Fig. 1c). Lowest surface δ^18^O_ara_ values (~−1.5‰) occurred just north of the equator at 10° N, in the same area where temperature was highest and salinity lowest. From this minimum, surface δ^18^O_ara_ increased continuously to the north and to the south until highest values were reached in the southernmost part of the transect (stations 62 and 66: ~+2‰). The distribution of δ^13^C_DIC_ in surface waters also exhibited latitudinal zonation, with highest values (~+2‰) occurring at the equator around 0° W and in the Western Atlantic south of 40° S (Fig. 1d). From the equator, surface values gradually decreased towards both the north and south with lowest surface δ^13^C_DIC_ values in the North Eastern Atlantic.

### δ^18^O in the water column and in pteropod shells

**Figure 2:**
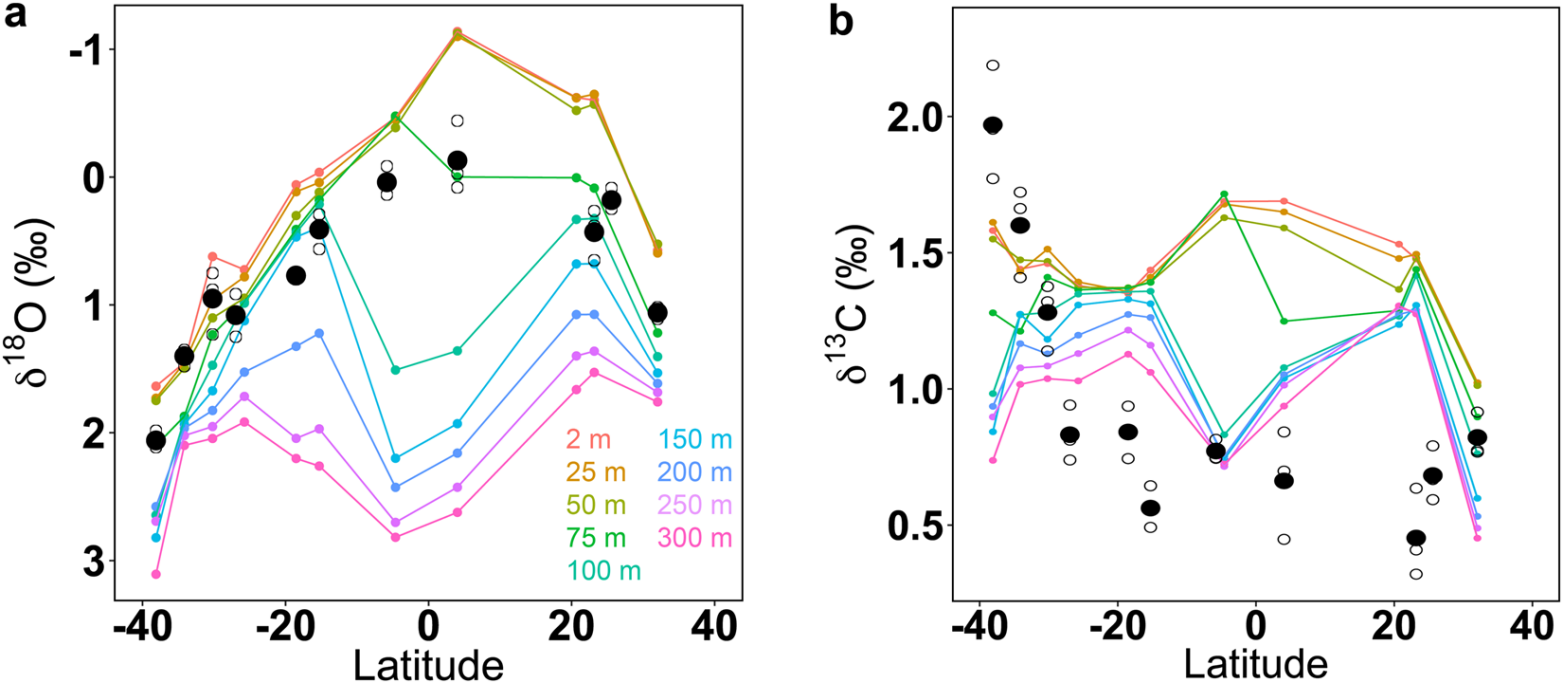
Latitudinal record of δ^18^O_ptero_ and δ^13^C_ptero_ in shells of *Heliconoides inflatus* pteropods. (**a**) δ^18^O_ptero_ (inversed y-scale) and (**b**) δ^13^C_ptero_ along a meridional transect in the Atlantic Ocean. Measurements on individual shells are shown (open circles) and closed circles represent averaged values. δ^18^O_ara_ and δ^13^C_DIC_ isopleths for different depths in the water﻿column are shown by colored lines as indicated by the legend.

Latitudinal variations in calculated δ^18^O_ara_ for equilibrium aragonite values at different depths in the water﻿column reflect strong thermal stratification between 20° N and 20° S, with a maximum in stratification around the equator (4° N), where values ranged from about −1.1‰ at the surface to +2.6‰ at 300 m depth (Fig. 2a). In comparison, δ^18^O_ara_ in the southern temperate region, e.g. at 34° S (station 62), was less variable across the water column (from +2.1‰ at the surface to +1.5‰ at 300 m depth).

Individual pteropod δ^18^O_ptero_ values varied with latitude (see Table 2, S2 and Fig. 2a): the lowest values (minimum: −0.44‰) occurred at the equator and increased towards higher latitudes (maximum: +2.11‰). Variability between specimens at each station was close to instrument precision, with an average standard deviation of 0.14 (range 0.05–0.28‰). The pteropod oxygen isotopic composition measured here was comparable to the study of Juranek and coworkers^12^ on the same species (+0.15 to +2.04‰), in which specimens from a one year-sediment trap in the Sargasso Sea were analyzed. Another study, however, reported more depleted oxygen isotopic values (approximately by 0.8‰)^16^ for the same species from the Sargasso Sea, which likely derived from low temperature ashing of the samples before isotopic measurement^12^.

**Table 2:** Average δ^18^O_ptero_ and δ^13^C_ptero_ (in ‰) based on measurements of individual pteropod shells (N = number of shells measured; SD = Standard Deviation, *n.a.* = not available, as n=2﻿).

CTD Station	Latitude	Longitude	N	δ^18^O_**ptero**_	SD	δ^13^C_**ptero**_	SD
15	32.02	−30.74	3	1.06	0.05	0.82	0.08
23	23.16	−40.60	3	0.43	0.20	0.45	0.16
25	20.57	−38.59	3	0.18	0.09	0.68	0.10
37	4.03	−26.47	3	−0.13	0.28	0.66	0.20
42	−4.62	−25.00	3	0.04	0.11	0.77	0.04
48	−15.29	−25.05	3	0.41	0.14	0.56	0.08
50	−18.52	−25.10	2	0.77	*n.a.*	0.84	*﻿n.a.*
56	−25.75	−24.99	3	1.08	0.17	0.83	0.10
60	−30.20	−27.92	3	0.95	0.25	1.28	0.12
62	−34.15	−33.49	3	1.40	0.07	1.60	0.17
66	−38.11	−39.33	3	2.06	0.07	1.97	0.21

**Figure 3:**
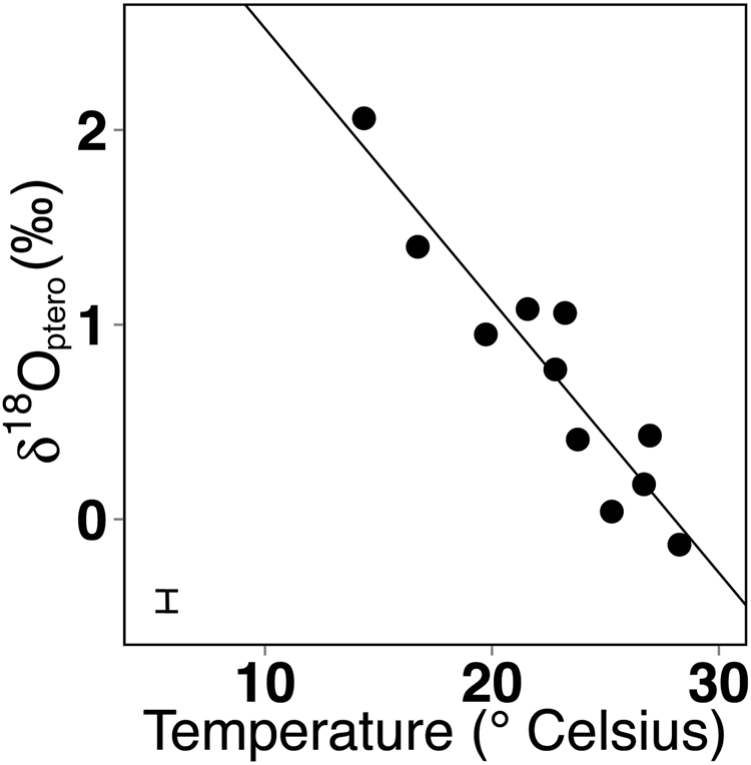
δ^18^O_ptero_ versus temperature at 50 m depth. We observed a significant negative relationship between δ^18^O_ptero_ and temperature (p < 0.05; R^2^ = 0.86): δ^18^O_ptero_ **= **−0.140 (±0.018) * Temperature + 3.919 (±0.404). Values in brackets indicate the standard error. Average standard deviation is depicted by the error bar in the lower left corner.

Average pteropod δ^18^O_ptero_ values were similar to those calculated near the 75m-depth isopleth of δ^18^O_ara_ (Fig. 2a), with individual measurements corresponding to values typically found between the surface and a maximum depth of 150 m. This result was also illustrated by the strong positive relationship between pteropod δ^18^O_ptero_ and δ^18^O_ara_ in the upper 75 m of the water column (p < 0.05; R^2^ = 0.89 to 0.91; Table 3, top). Likewise, we found a strong linear relationship between temperature and pteropod δ^18^O_ptero_ (p < 0.05; R^2^ = 0.87 to 0.86) in the upper 75 m of the water column (Fig. 3, Table 3, top). δ^18^O_ptero_ also showed a positive, but weaker relationship to carbonate ion concentration (p < 0.05, R^2^ = 0.64) at the surface, while no statistically significant correlation with salinity was found.

**Table 3:** Linear relationships between average pteropod shell δ^18^O_ptero_ (top), δ^13^C_ptero_ (middle and bottom) and water parameters: carbonate ion concentration (μmol/kg), temperature (° Celsius), salinity, Chlorophyll *a* (Chl *a* *)*, δ^18^O_ara_ (‰) and δ^13^C_DIC_ (‰). Regressions were performed against parameters at specific depths; the adjusted R^2^ is reported when p < 0.05. *n*.*s*. indicates non-significant linear regressions (p > 0.05). Linear regressions for correlations with water parameters at 50 m depths are listed above the tables, values in parentheses are the respective standard errors.

δ^18^O (all stations)					
δ^18^O_ptero_ = −0.022 (±0.006) *Carbonate + 5.661 (±1.292)					
δ^18^O_ptero_ = −0.140 (±0.018) *Temperature + 3.919 (±0.404)					
δ^18^O_ptero_ = 0.660 (±0.078) *δ^18^O_ara_ + 0.532 (±0.073)					
**Depth (m)**	**Carbonate**	**Temperature**	**Salinity**	**δ** ^**18**^ **O**_**ara**_	
2	0.64	0.87	*n*.*s*.	0.91	
25	0.62	0.87	*n*.*s*.	0.90	
50	0.58	0.86	*n*.*s*.	0.88	
75	*n*.*s*.	0.86	*n*.*s*.	0.89	
100	*n*.*s*.	*n*.*s*.	*n*.*s*.	0.33	
150	*n*.*s*.	*n*.*s*.	*n*.*s*.	*n*.*s*.	
200	*n*.*s*.	*n*.*s*.	*n*.*s*.	*n*.*s*.	
250	*n*.*s*.	*n*.*s*.	*n*.*s*.	*n*.*s*.	
300	*n*.*s*.	*n*.*s*.	*n*.*s*.	*n*.*s*.	
**δ** ^**13**^ **C (all stations)**					
**δ** ^**13**^ **C**_**ptero**_ = −0.0198 (±0.002) *Carbonate + 5.306 (±0.410)					
**δ** ^**13**^ **C**_**ptero**_ = −0.101 (±0.013) *Temperature + 3.239 (±0.300)					
**δ** ^**13**^ **C**_**ptero**_ = −0.474 (±0.119) *Salinity + 18.241 (±4.327)					
**Depth (m)**	**Carbonate**	**Temperature**	**Salinity**	**Chl**. ***a***	**δ** ^**13**^ **C**_**DIC**_
2	0.88	0.87	0.41	*n*.*s*.	*n*.*s*.
25	0.90	0.88	0.51	*0*.*80*	*n*.*s*.
50	0.92	0.86	0.60	*0*.*73*	*n*.*s*.
75	0.67	0.86	0.71	*n*.*s*.	*n*.*s*.
100	*n*.*s*.	0.70	0.58	*n*.*s*.	*n*.*s*.
150	*n*.*s*.	0.50	0.49	*n*.*s*.	*n*.*s*.
200	*n*.*s*.	0.42	0.36	*n*.*s*.	*n*.*s*.
250	*n*.*s*.	*n*.*s*.	*n*.*s*.	*n*.*s*.	*n*.*s*.
300	*n*.*s*.	*n*.*s*.	*n*.*s*.	*n*.*s*.	*n*.*s*.
**δ** ^**13**^ **C (stations with** ^**13**^ **C enrichment (#60, 62 & 66) excluded)**					
**δ** ^**13**^ **C**_**ptero**_ = −0.010 (±0.002) *Carbonate + 3.067 (±0.386)					
**Depth (m)**	**Carbonate**	**Temperature**	**Salinity**	**Chl**. ***a***	**δ** ^**13**^ **C**_**DIC**_
2	0.68	*n*.*s*.	*n*.*s*.	*n*.*s*.	*n*.*s*.
25	0.82	*n*.*s*.	*n*.*s*.	*n*.*s*.	*n*.*s*.
50	0.84	*n*.*s*.	*n*.*s*.	*n*.*s*.	*n*.*s*.
75	*n*.*s*.	*n*.*s*.	*n*.*s*.	*n*.*s*.	*n*.*s*.
100	*n*.*s*.	*n*.*s*.	*n*.*s*.	*n*.*s*.	*n*.*s*.
150	*n*.*s*.	*n*.*s*.	*n*.*s*.	*n*.*s*.	*n*.*s*.
200	*n*.*s*.	*n*.*s*.	*n*.*s*.	*n*.*s*.	*n*.*s*.
250	*n*.*s*.	*n*.*s*.	*n*.*s*.	*n*.*s*.	*n*.*s*.
300	*n*.*s*.	*n*.*s*.	*n*.*s*.	*n*.*s*.	*n*.*s*.

### δ^13^C in the water column and in pteropod shells

**Figure 4:**
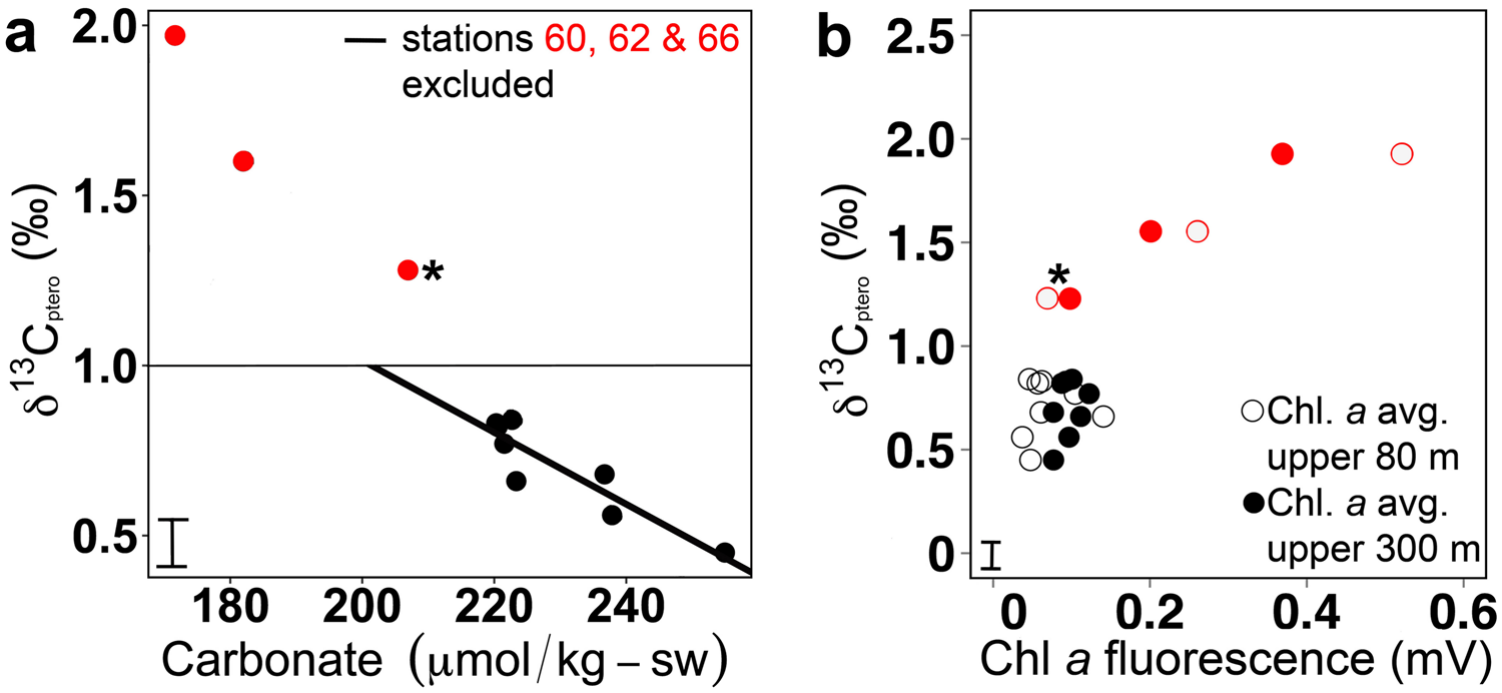
Relationship between δ^13^C_ptero_ and water parameters. (**a**) δ^13^C_ptero_ versus average carbonate ion concentration in the upper 80 m. The carbonate ion effect on δ^13^C_ptero_ is seen in the negative relationship between carbonate ion concentration and δ^13^C_ptero_ (solid line). Due to high phytoplankton productivity related ^13^C enrichment, stations 60, 62 & 66 were removed; the horizontal line indicates the application limit of the calibration (see text). (**b**) δ^13^C_ptero_ versus chlorophyll *a* (Chl. *a*) fluorescence. Stations 60, 62 & 66 marked in red and station 60 is marked by an asterisk (see Discussion). Error bars in the lower left corner indicate average standard deviation.

**Figure 5:**
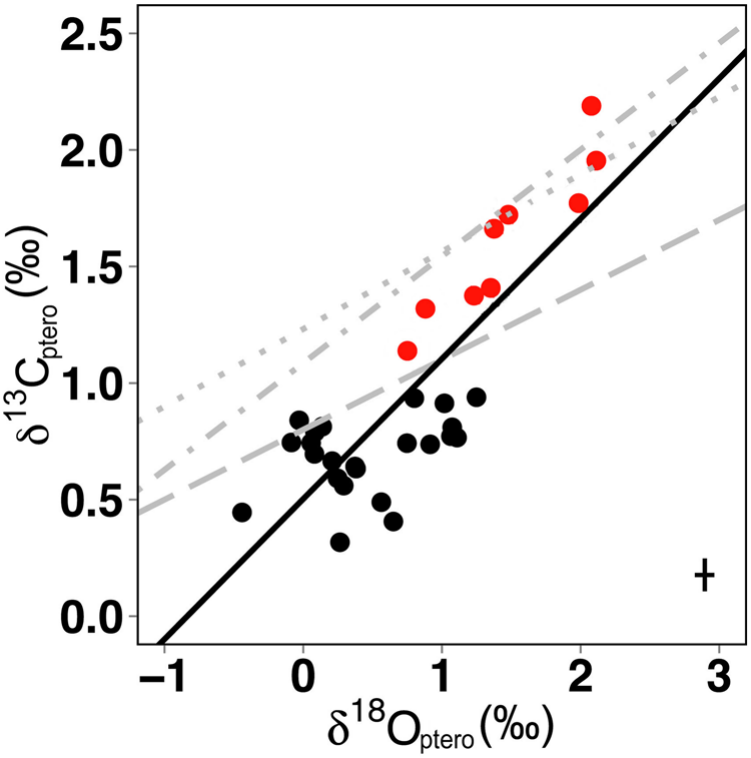
Linear relationships between pteropod shell δ^13^C_ptero_ and δ^18^O_ptero_. Our data: δ^13^C_ptero_ = 0.54 * δ^18^O_ptero_ + 0.50 with p = 0.001078 and R^2^ = 0.68, as denoted by the black line. Grey lines indicate relationships found for the same species by other researchers: dashed (R^2^ = 0.3, Sargasso Sea)^16^, dot-dashed (R^2^ = 0.2, Cape Blanc)^19^, dotted (R^2^ = 0.5, Cape Blanc)^18^. Small black filled circles denote our measurements (triplicates, station 50 in duplicates) from all stations, with measurements from the three southernmost stations (60, 62, 66) depicted in red. The cross in the lower right corner indicates standard deviation (for δ^13^C_ptero_ and δ^18^O_ptero_).

Pteropod shell δ^13^C_ptero_ showed little variation in samples that were collected between 32° N and 26° S (+0.77‰ ± 0.23‰), however, δ^13^C_ptero_ increased sharply with mean values of +1.28, +1.60, and +1.97‰ at the southernmost stations 60, 62, and 66, respectively (Fig. 2b, Table 2 and S2). Calculated variation of δ^13^C_DIC_ in the water column was highest at low latitudes, where the variability of δ^13^C_ptero_ in pteropod shells was very low (Fig. 2b). Pteropod shell δ^13^C_ptero_ at most stations was lower than calculated δ^13^C_DIC_ in the water column. This result was previously observed in pteropod shells that calcified in shallow waters^12^, and was attributed to the carbonate ion effect, where higher carbonate ion concentrations caused lower δ^13^C_ptero_ values than in equilibrium with δ^13^C_DIC_ (see Discussion). We find the strongest linear relationship between pteropod δ^13^C_ptero_ and carbonate ion concentration in the upper water column (Fig. 4a), with a negative regression of δ^13^C_ptero_ = −0.02 * Carbonate (50 m) + 5.31 (p < 0.05, R^2^ = 0.92; Table 3, middle). Furthermore, we observe linear relationships between pteropod δ^13^C_ptero_ and temperature and salinity, but none with δ^13^C_DIC_ (Table 3, middle). Pteropod shells from the southernmost stations have high δ^13^C_ptero_ values, where phytoplankton standing stock was high in surface waters (see Discussion below and Fig. 4b). We observe a significant positive relationship between δ^13^C_ptero_ and δ^18^O_ptero_ in pteropod shells (p < 0.05, R^2^ = 0.68; Fig. 5). Interestingly, this relationship is mostly influenced by measurements performed on shells from the three southernmost stations (60, 62 & 66; indicated by open circles in Fig. 5): omitting these stations renders the relationship statistically non-significant (p > 0.05). Comparison of our results to previous findings on the same species^16,18,19^ revealed that the slope was similar to prior work (Fig. 5). However, the δ^13^C_ptero_ values presented here cover a broader range than observed in previous studies that were located in environments with less oceanographic variation (e.g., sediment trap studies, Sargasso Sea).

## Discussion

Deriving past ocean temperature and chemistry from fossil pteropod shells provides a wealth of information about past climate change events. The present study shows that the species *H*. *inflatus* is well suited for paleo-reconstructions, as the stable isotopic composition of their shells can be used to track two climate change indicators: δ^18^O_ptero_ records temperature (Fig. 3) and carbonate ion concentration is traced by δ^13^C_ptero_ (Fig. 4a). Both proxies can be measured simultaneously on a single pteropod shell, making pteropods particularly promising new proxy carriers. Furthermore, we demonstrate that *H*. *inflatus* records latitudinal ranges of surface water parameters, as suggested previously from sediment trap studies at a local scale^12,16^. We confirm shallow water calcification of this species for a large area of the Atlantic Ocean (31° N to 38° S), which, in combination with its basin-wide distribution, renders *H*. *inflatus* an ideal candidate for proxy reconstructions.

Based on a comparison between the oxygen isotopic composition of pteropod shells and that of seawater, we observed no systematic latitudinal variation in calcification depth (Fig. 2a). Oxygen isotopic values for *H*. *inflatus* strongly correlated with δ^18^O_ara_ equilibrium values in the upper 75 m of the water column (Fig. 2a, Table 3, top). These findings corroborate observations from other studies on the same species in the Sargasso Sea, suggesting calcification from 50–250 m depths^12,16^. Such shallow calcification depths are in contrast to reported preferential occurrences of *H*. *inflatus* in deeper waters (﻿to 600 m) in the Sargasso Sea^17^. One possible explanation is that pteropods preferentially calcify near the surface, where calcification is energetically favored due to warmer temperatures, affecting calcium carbonate saturation. Furthermore, most chlorophyll *a*, and thus potential food resources, occurs in the upper water column, which may be another reason why *H*. *inflatus* calcifies in shallow waters.

Pteropod shells are produced over several months, therefore reflecting the sum of environmental conditions experienced throughout the animal’s life. Thus, single-shell measurements, as presented here, are an average of these conditions, with a bias toward the more recently calcified material, as this makes up the largest part of the shell^16^. Pteropods alter their depth habitat daily, seasonally, and ontogenetically^9,17^. Consequently, the aragonite of a single pteropod shell could have been produced across a range of depths. Accordingly, the estimated calcification depth of about 75 m may be the average of varying isotopic signatures from different calcification depths. However, several observations argue against this. First, we found no correlation of δ^18^O_ptero_ and temperature below 100 m. Second, other studies on the same species also found that *H*. *inflatus* mostly calcified in the upper water column, rather than in deeper waters^12,16^. The material of a single pteropod shell is probably also the product of several seasons, as the average life span of several pteropod species is on the order of one year^15^. Sediment trap material is ideal to study the effect of seasonality on the stable isotopic composition of pteropods. Two other studies^12,16^ found that δ^18^O_ptero_ measurements on seasonal *H*. *inflatus* samples from sediment traps usually correlate strongly with seasonal temperature variations in the water column. The absence of an offset in time between δ^18^O_ptero_ and δ^18^O_ara_ in these studies indicates that the bulk of the shell must have been precipitated within the few months prior to collection, making pteropods reliable recorders of surface water masses.

Carbon isotopic composition of pteropod shells was relatively invariant along the meridional transect in the Atlantic Ocean, with the exception of higher δ^13^C_ptero_ values at stations south of 30°S (60, 62 and 66; Fig. 4a). Phytoplankton standing stock (chlorophyll *a* concentration) was very high in surface waters (upper 75 m of the water column) in the south subtropical convergence province (stations 62 and 66, see ref.^23^). Photosynthesis actively removes the lighter carbon isotopes from the water column DIC reservoir, resulting in ^13^C_DIC_ enrichment. Since pteropods use ambient DIC to build their shells, δ^13^C_ptero_ will also be higher in regions with high rates of photosynthesis, as was observed at these southernmost stations (Fig. 4b). The positive relationship between δ^13^C_ptero_ and chlorophyll *a* at 25 and 50 m depth (Table 3, middle, p < 0.05, R^2^ = 0.80 and 0.73, respectively) also reflects the effect of photosynthesis on δ^13^C_ptero_. Additionally, *H*. *inflatus* specimens from southern temperate waters (south of 34°S) along a similar transect (AMT24) were reported to be morphologically distinct, having coarser and thicker shells than specimens from the rest of the transect, and thus may represent a distinct population or (sub)species (referred to as *H*. *inflatus* S)^24^. In these respects, station 60 (30.20 °S) should be regarded as a transitional station: it is located in the oligotrophic south Atlantic gyral province with low chlorophyll *a* concentrations and *H*. *inflatus* specimens that were morphologically similar to specimens from tropical and subtropical waters, but average δ^13^C_ptero_ in their shells was relatively high (Fig. 4b, station marked by asterisk). While the (calcium) isotopic signature of foraminiferal sub-species has been demonstrated to be the same^25^, small trace element compositional differences can still be caused by depth habitat preferences of different sub-species^26^. All pteropods analyzed here calcify in the same water depth (upper 75 m, Fig. 2a), allowing us to assume that the potentially different (sub) species have similar isotopic signatures under the same environmental conditions. Pteropod δ^13^C_ptero_ did not show much variation between 32° N and 26° S (Fig. 2b), where chlorophyll *a* concentration was much lower (Fig. 4b). Apparently, photosynthesis by these low phytoplankton concentrations did not cause a ^13^C enrichment of the DIC pool, explaining the relatively low δ^13^C_ptero_ values of the pteropods across most of the transect (average + 0.70‰). However, as we observe no correlation between chlorophyll *a* fluorescence and δ^13^C_ptero_ in the majority of the pteropod shells analyzed (Fig. 4b), other influences on δ^13^C_ptero_ should be explored, such as the carbonate ion effect. This effect was reported in foraminifera^13,27^ and in pteropods^12^, and describes the inverse relationship between carbonate ion concentration and δ^13^C_ptero_, which is also apparent in our results (Fig. 4a, R^2^ = 0.94, p < 0.05). For the southernmost stations, it is impossible to disentangle the effects of ^13^C enrichment via photosynthesis and the carbonate ion effect on pteropod δ^13^C_ptero_ (Table 3). We therefore performed linear regressions on δ^13^C_ptero_ and water column parameters while excluding stations 60, 62, and 66 (Table 3, bottom). Excluding these three stations clearly demonstrates the carbonate ion effect on δ^13^C_ptero_ (Table 3, bottom, R^2^ = 0.84, p < 0.05) in the upper water column of an area where no ^13^C enrichment is occurring (no effect of chlorophyll *a* on δ^13^C_ptero_; Table 3, bottom, p > 0.05).

Our study shows that the pteropod species *H*. *inflatus* calcifies across a number of oceanographic provinces in the Atlantic at the same, shallow depth (upper 75 m of the water column, Fig. 2a, Table 3), making these pteropod shells good recorders of surface water masses. Correlations between stable isotopic composition of shells and parameters of the water column indicate that *H*. *inflatus* shells are good proxy carriers for temperature and carbonate ion reconstructions with the following regressions (for values at 50 m depth):

1 $$temperature=\frac{{\delta }^{18}{O}_{{\rm{p}}{\rm{t}}{\rm{e}}{\rm{r}}{\rm{o}}}-3.919\,(\pm 0.404)}{-0.140\,(\pm 0.140)}$$

with p < 0.05, R^2^ = 0.86 (Table 3, top), and

2 $$carbonate\,ion\,concentration=\frac{{\delta }^{13}{C}_{{\rm{p}}{\rm{t}}{\rm{e}}{\rm{r}}{\rm{o}}}-3.067\,(\pm 0.386)}{-0.010\,(\pm 0.002)}$$

with p < 0.05, R^2^ = 0.92 (Table 3, bottom), only valid for δ^13^C_ptero_ < 1‰ (see Discussion below).

The uncertainty in the estimations for temperature (equation 1) and carbonate ion concentration (equation 2) based on an error propagation calculation sums to an error of 17% and 25%, respectively, assuming a measurement precision of 0.09‰ and 0.05‰ for the measurements of δ^18^O_ptero_ and δ^13^C_ptero_, respectively. Please note that the regressions reported above (equations 1 and 2) have been derived from different datasets. While the δ^18^O_ptero_-temperature regression (equation 1) includes all stations, the δ^13^C_ptero_-carbonate ion regression (equation 2) is not valid for high productivity waters (here 31° S to 38° S), as the δ^13^C_ptero_ in these regions may be influenced by ^13^C enrichment (see Discussion above). Consequently, the calibration (equation 2) should only be used on δ^13^C_ptero_ values < 1‰ limiting the resolvable carbonate ion concentration to values of 200 μmol/kg-sw or higher.

*Heliconoides inflatus* is a pteropod species that not only occurs in the Atlantic, but has a circumglobal distribution in tropical and subtropical waters (including the Caribbean, Mediterranean and Indo-Pacific). Therefore, it is a good proxy carrier to assess surface water variations over paleo-timescales worldwide. *Heliconoides* is the oldest known pteropod genus in the fossil record (72–79 million years ago (mya)^28^), and the species *H*. *inflatus* has been described to occur at least since the early Miocene (Aquitanian) from the Aquitaine and North Sea basins (23.03–20.44 mya^29^ and pers. comm. Janssen 2017). One limitation on the application of this new ﻿proxy is the occurrence of well-preserved pteropod shells in sediments, confined to waters above the lysocline of aragonite. However, there are a number of sediment cores available in which *H*. *inflatus* is abundant and where the calibrations reported here can be applied. The CAR-MON2 core^22^ would be an ideal candidate from the Caribbean Sea, as it contains *H*. *inflatus* in great abundance. The core spans the last 250,000 years, and the associated changes in the ocean’s temperature and carbonate ion concentration during glacial/ interglacial cycles are well resolvable by the proxy calibrations reported here. This holds true even under the restriction of the δ^13^C_ptero_ calibration, as surface carbonate ion concentration in the Caribbean Sea has been >250 μmol/kg-sw for the last 100, 000 years^30^.

## Methods

### Pteropod collection

Bulk zooplankton was collected on the Atlantic Meridional Transect Cruise 22 (AMT22) between 10/19/2012 and 11/16/2012. Oblique tows were conducted with bongo nets (200 µm, 333 µm), towed between on average 361 m depth and the sea surface. Pteropods were collected from a total of 11 stations, between 31° N to 38° S latitude, in the pre-dawn hours (Table S1). After collection, pteropods were immediately fixed in pure ethanol (96–99%), which was renewed within 12–24 hours of collection. Specimens were stored at −20 °C until analysis.

### Measurements of stable isotopes (δ^18^O and δ^13^C) of pteropod shells

Pteropods (*H*. *inflatus*) within a narrow size range (800–1200 μm shell width) were removed from ethanol and dried at room temperature. All individual shells were weighed on a microbalance to ensure sufficient material for isotopic analysis (sample mass 120 ± 60 (1 SD) μg on average). Shells were broken to allow removal of the soft-tissue. All shell pieces were collected, triple rinsed with ultrapure water, dried at room temperature and weighed. The isotopic composition was analyzed at the Leibniz Laboratory for Radiometric Dating and Stable Isotope Research (Kiel University, Germany) using a Kiel IV carbonate preparation device connected to a ThermoScientific MAT 253 mass spectrometer. The aragonitic shells were reacted with 100% phosphoric acid (H_3_PO_4_) under vacuum at 75 °C, and the evolved carbon dioxide gas was analyzed eight times for each individual sample. All values are reported in the Vienna Pee Dee Belemnite notation (VPDB) relative to NBS19. Precision of all different laboratory internal and international standards (NBS19) is ≤±0.05‰ for δ^13^C and ≤±0.09‰ for δ^18^O values. For notations related to shell chemistry, see Table 1.

Isotope values are reported in standard δ notation where:

3 $${\delta }^{18}O=[\frac{\frac{{}^{18}O}{{}^{16}O}{\rm{sample}}}{\frac{{}^{18}O}{{}^{16}O}{\rm{standard}}}-1]\,\ast \,1000$$

4 $${\delta }^{13}C=[\frac{\frac{{}^{13}C}{{}^{12}C}{\rm{sample}}}{\frac{{}^{13}C}{{}^{12}C}{\rm{standard}}}-1]\,\ast \,1000$$

Averaged values of triplicate measurements are reported, except for station 50, where measurements were only possible in duplicate (Tables 2 and S2).

### Seawater parameters: temperature, salinity, carbonate chemistry and chlorophyll *a*

Seawater temperature, salinity, and chlorophyll *a* concentrations in the upper 500 m of the water column were obtained by conductivity-temperature-depth (CTD) casts (Sea-Bird Electronics, models: ocean logger, SBE45, 9plus) and Chelsea MKIII Aquatracka Fluorometer, respectively. Sensors were calibrated and data archived by the British Oceanographic Data Centre (BODC). Discrete seawater samples taken from Niskin bottles were used to measure pH, TA (total alkalinity) and DIC. In order to calibrate the CTD chlorophyll fluorometer, discrete Chlorophyll *a* samples were analyzed fluorometrically following standard acetone extraction^31^. Briefly, discrete chlorophyll *a* samples were filtered through GF/F filters (0.7 μm) and placed in acetone for 18–36 hours before fluorescence was measured on a Turner Designs AU10 fluorometer.

pH was measured (~11 samples per station) spectrophotometrically according to Clayton and Byrne^32^. TA was measured at selected depths, including ~3 samples per station (e.g. at approximately 300, 100 and 2 m depth for station 15), and analyzed by open-cell-titration^33^. TA measurements were related to salinity and temperature^34^ according to the polynomial described by Lee and colleagues^35^, and were subsequently used to estimate TA at all depths. TA and pH were used to calculate the complete C-system (DIC, bicarbonate, carbonate, Omega and Revelle Factor at all depths) using the CO2SYS software^36^. These calculations were consistent with measured DIC (at selected depths, ~3 depths per station, same depths as TA measurements) and surface pCO_2_ (CO_2_ partial pressure) measured continuously every 20 minutes^34^.

### Seawater composition in the sampling area

In order to characterize environmental controls on pteropod stable isotopic composition (δ^18^O_ptero_ and δ^13^C_ptero_), seawater δ^18^O_ara_ and δ^13^C_DIC_ isopleths were calculated (surface to 300 m). The salinity- δ^18^O_SW_ (seawater: sw) calibrations from Le Grande and Schmidt^37^ for Atlantic provinces were used, with CTD-derived salinity (S) as an input parameter: North Atlantic (δ^18^O_SW_ = 0.55*S − 18.98), Tropical Atlantic (δ^18^O_SW_ = 0.15*S − 4.61) and Southern Atlantic (δ^18^O_SW_ = 0.51*S −17.40). Thereafter, δ^18^O_ara_ was calculated from temperature and δ^18^O_SW_ according to: δ^18^O_ara_ = (T−20)/(−4.42) + δ^18^O_SW_^7^, using temperature (T) from CTD measurements. There was no suitable calibration for the correlation of δ^13^C_DIC_ and DIC, therefore we used the GLODAP data set (http://cdiac.ornl.gov/oceans/GLODAPv238) to calculate linear regressions between δ^13^C_DIC_ and DIC. We defined six oceanic provinces according to latitude (45°N-30°N, 30°N-15°N, 15°N -0°N, 0°S-23°S, 23°S-30°S, 30°S-45°S) and used all available data in the upper 105 m between 10° E and 60° E, yielding six regressions for the relationship between δ^13^C_DIC_ and DIC (Table S3). The uncertainty in these calculations based on an error propagation calculation assuming a DIC concentration of 2200 (±10) μmol/kg sums to an average error of 22% for the δ^13^C_DIC_ estimation and to an error of 5% in the δ^18^O_SW_ estimation, when assuming a salinity of 35 (±0.1) and temperature of 24 (±0.1) ° C. These values have been calculated using the standard errors listed in Table S3 and the respective publications^7,37^. Data from the World Ocean Database (WOD)^38^ and GLODAP^39^ were used to generate surface distribution maps of the Atlantic for temperature, salinity and seawater δ^18^O_ara_ and δ^13^C_DIC_. Plots present average values from October through November in order to obtain a representation of the typical surface distribution of these parameters during the period of the cruise (10/13/2012 to 11/19/2012). The WOD^38^ data collection contained all surface data available from 1986 to 2011, and the GLODAP^39^ data collection contained all data from 1972 to 2011.

### Statistical analyses

To test the effect of temperature, salinity, carbonate ion concentration, chlorophyll *a* concentration, δ^18^O_ara_ and δ^13^C_DIC_ on pteropod shell isotopic composition (δ^18^O_ptero_ and δ^13^C_ptero_), linear regressions were calculated for specific depths (2, 25, 50, 75, 100, 200, 250, 300 m). Temperature, salinity and chlorophyll *a* were taken from the CTD casts. Carbonate ion concentration was interpolated to these depths, while δ^18^O_ara_ and δ^13^C_DIC_ were calculated at these depths (see above).

### Data availability

All data generated or analyzed during this study are included in this published article (and its Supplementary Information files).

## Electronic supplementary material

Supplementary Data
