# Supplementary material for: Pteropods are excellent recorders of surface temperature and carbonate ion concentration
Source: Sci Rep. 2017 Oct 3;7:12645. doi: 10.1038/s41598-017-11708-w (PMC5626693; doi:10.1038/s41598-017-11708-w)
Supplement: Supplementary file 1 — Supplementary Data [file 41598_2017_11708_MOESM1_ESM.pdf]

SUPPLEMENTARY INFORMATION

Pteropods are excellent recorders of surface temperature and carbonate ion concentration

N. Keul[1*], K.T.C.A. Peijnenburg[2,3], N. Andersen[4], V. Kitidis[5], E. Goetze[6], R. Schneider[1]

[1] *Institute of Geosciences, Christian-Albrechts-Universität zu Kiel, Ludewig-Meyn-Str.10, 24118 Kiel, Germany*

[2] *Naturalis Biodiversity Center, P.O. Box 9517, 2300 RA Leiden, The Netherlands*

[3] *Institute for Biodiversity and Ecosystem Dynamics (IBED), University of Amsterdam, P.O. Box 94248, 1090 GE Amsterdam, The Netherlands*

[4] *Leibniz-Labor für Altersbestimmung und Isotopenforschung, Christian-Albrechts-Universität zu Kiel, Max-Eyth-Str.11-13, 24118 Kiel, Germany*

[5] *Plymouth Marine Laboratory, Plymouth, United Kingdom*

[6] *Department of Oceanography, University of Hawai'i at Mānoa, 1000 Pope Road, Honolulu, HI 96822, USA*

* Correspondence to nina.keul@gmail.com.

Table S1: Sampling information of *H. inflatus* pteropods used in this study.

Plankton Station/ Cruise	Corresponding CTD Station (Table 2)	Latitude	Longitude	Date	Local time (start tow)	Tow duration (min)	Max depth of tow (m)
15/AMT22	15	32.00	-30.74	10/19/12	2:41	47	363
23/AMT22	23	23.15	-40.63	10/23/12	2:43	48	361
25/AMT22	25	20.40	-38.61	10/24/12	2:45	47	336
37/AMT22	37	4.05	-26.46	10/30/12	2:42	53	388
43/AMT22	42	-4.62	-25.02	11/2/12	2:42	48	488
49/AMT22	48	-15.29	-25.07	11/5/12	2:39	48	322
51/AMT22	50	-18.49	-25.10	11/6/12	2:42	48	373
57/AMT22	56	-25.73	-24.99	11/10/12	2:24	47	350
60/AMT22	60	-30.17	-27.91	11/12/12	2:25	48	323
62/AMT22	62	-34.12	-33.50	11/14/12	2:39	48	376
66/AMT22	66	-38.08	-39.31	11/16/12	2:43	50	292

Table S2: Individual and average (avg.) $\delta^{18}O_{ptero}$ and $\delta^{13}C_{ptero}$ based on measurements of individual pteropod shells (ind. meas.), including the internal measurement error (internal error) and standard deviation (SD).

CTD Station	$\delta^{18}O_{ptero}$ ind. meas.	$\delta^{18}O_{ptero}$ internal error	$\delta^{13}C_{ptero}$ ind. meas.	$\delta^{13}C_{ptero}$ internal error	$\delta^{18}O_{ptero}$ avg.	$\delta^{18}O_{ptero}$ SD	$\delta^{13}C_{ptero}$ avg.	$\delta^{13}C_{ptero}$ SD
15	1.02	0.03	0.91	0.01				
	1.11	0.02	0.77	0.01	1.06	0.05	0.82	0.08
	1.07	0.02	0.77	0.01				
23	0.38	0.04	0.63	0.02				
	0.65	0.03	0.41	0.02	0.43	0.20	0.45	0.16
	0.27	0.03	0.32	0.01				
25	0.09	0.03	0.79	0.01				
	0.25	0.01	0.59	0.01	0.18	0.09	0.68	0.10
	0.21	0.05	0.67	0.02				
37	0.08	0.02	0.70	0.00				
	-0.03	0.03	0.84	0.01	-0.13	0.28	0.66	0.20
	-0.44	0.01	0.45	0.01				
42	0.06	0.02	0.74	0.01				
	-0.09	0.03	0.75	0.01	0.04	0.11	0.77	0.04
	0.14	0.02	0.81	0.01				
48	0.56	0.02	0.49	0.01				
	0.37	0.03	0.64	0.01	0.41	0.14	0.56	0.08
	0.29	0.02	0.56	0.01				
50	0.75	0.02	0.74	0.01	0.77	0.03	0.84	0.10
	0.80	0.02	0.94	0.01				
56	1.25	0.02	0.94	0.00				
	0.92	0.02	0.74	0.01	1.08	0.17	0.83	0.10
	1.07	0.02	0.81	0.01				
60	0.88	0.01	1.32	0.02				
	1.23	0.03	1.38	0.02	0.95	0.25	1.28	0.12
	0.75	0.04	1.14	0.01				
62	1.38	0.03	1.66	0.01				
	1.48	0.01	1.72	0.01	1.40	0.07	1.60	0.17
	1.35	0.02	1.41	0.01				
66	1.98	0.01	1.77	0.00				
	2.11	0.01	1.95	0.01	2.06	0.07	1.97	0.21
	2.08	0.01	2.19	0.00				

Table S3: Regional δ13C- DIC Relationships in the Atlantic Ocean determined for this study and basic statistics. n= total points, SE= standard error.

Latitude	n	Slope	SE Slope	Intercept	SE Intercept	R^2
30°N - 45°N	94	-0.0105	0.0012	22.947	2.537	0.44
15°N - 30°N	90	-0.0031	0.0006	7.934	1.166	0.35
0°N - 15°N	54	-0.0029	0.0004	7.393	0.886	0.45
0°S - 23°S	69	-0.0054	0.0005	12.735	0.987	0.65
23°S - 30°S	38	-0.0097	0.0023	21.641	4.850	0.31
30°S - 45°S	57	-0.0082	0.0018	18.503	3.798	0.25

Table S4: Matrix showing correlation of water parameters in the upper 300 m of the water column along the cruise track. Pearson's R is shown.

	$\delta^{13}C_{DIC}$	carbonate ion concentration	temperature	salinity
$\delta^{18}O_{ara}$	-0.68	-0.89	-0.98	-0.72
$\delta^{13}C_{DIC}$		0.65	0.63	0.31
carbonate ion concentration			0.92	0.81
temperature				0.79
